# Seismic Risk Classification of Building Clusters Using MST Clustering and UAV Remote Sensing

**DOI:** 10.3390/s25030744

**Published:** 2025-01-26

**Authors:** Xianteng Wang, Xue Li, Zhumei Liu, Zihao Wu, Yike Xie, Zijie Han

**Affiliations:** Key Laboratory of Earthquake Geodesy, Institute of Seismology, China Earthquake Administration, Wuhan 430071, China; wangxt0724@126.com (X.W.); 15337136270@163.com (Z.H.)

**Keywords:** UVA remote sensing, buildings, MST clustering, structure type classification

## Abstract

The fundamental attribute that is essential for the seismic capacity assessment of houses is the building structure type. Conventionally, remote sensing assessment of the seismic capacity for houses has been based on the image features of individual houses, instead of the spatial similarity between them. To enhance the classification accuracy of house structure types, this work proposes a minimum spanning tree (MST) house clustering structure type classification method based on the spatial similarity of houses. First, the method employs the geometric characteristics of residential buildings to calculate the Gestalt factor that characterizes the visual distance. Subsequently, a Delaunay triangular mesh is constructed to create a proximity map between the houses, with the MST generated using visual distance as the weighting factor. Then, the spatial proximity similarity of house clusters is obtained through pruning. Finally, a support vector machine is employed to categorize the architectural structure of the housing complex, viz., simple houses, brick–concrete houses, and frame houses. This classification is based on the geometric, textural, height, and spatial distribution characteristics of the houses. We have conducted a remote sensing classification experiment of house structure types with Zhushan County, Hubei Province as the study area. The results show that the MST clustering method improves the classification accuracy of brick–concrete houses to 95.4% and the classification accuracy of simple houses to 93.4%. Compared to the single-family-based classification method of building structure types, the classification accuracy of frame-structure buildings is improved to 87%. The Kappa coefficient increased to 0.89. This study significantly improves the classification accuracy of building structure types by introducing spatial similarity. Furthermore, it shows the potential for spatial similarity in classifying building structure types.

## 1. Introduction

Building collapse is a major cause of casualties and property damage during earthquakes [1]. In recent years, seismic vulnerability analysis has become an important method for evaluating the seismic performance of buildings. Constructing seismic vulnerability curves can quantify the probability of damage to buildings under various seismic intensities [2,3]. However, existing vulnerability analysis methods heavily rely on information related to structural types, and most focus on specific building categories, such as nuclear power plants or bridge piers. Although these studies provide valuable insights for vulnerability analysis, they require high accuracy in classifying building structural types and depend significantly on the availability of relevant data. The type of housing structure is the key information required for assessing the seismic capacity of houses, and the seismic capacity of houses with different types of structures varies widely [4]. The traditional housing structure type survey is generally carried out by a field survey. Although the survey data are comprehensive and highly accurate, they limit the survey efficiency and make it difficult to carry out on a large scale, owing to the high professional threshold [5].

Recently, several works have applied remote sensing technology in researching the classification of building structure types. Geiß et al. [6] have proposed a method of building structure type estimation that combines in situ observations and multi-source remote sensing data. The classification results revealed the correlation between features such as the building shape, height, and spectrum and the type of seismic-resistant structure of a house. Yu et al. [7] have validated the capacity of UAV image data to discern the structural typologies of Liaoning farmhouses through empirical investigation, thus enhancing the precision of remote sensing analysis for these structures to 75%, based on the proportion of regional typologies and the characteristics of the images captured. Du et al. [8] have developed a methodology for classifying the building structures based on the fusion of multiple features extracted from the UAV remote sensing images. This approach integrates the information from multiple sources, including texture, spectral characteristics, and height data, to enhance the accuracy of house structure type recognition.

Recent studies based on remote sensing for the classification and identification of building structure types primarily focus on single-house buildings with structure types classified based on the spectral, textural, and height characteristics of the houses in question [9], without considering the spatial relationship between the houses. However, the spatial distribution of urban houses is frequently analogous in neighboring geographic areas, owing to the combined influence of socioeconomic, cultural, and natural factors [10,11]. Based on the spatial cognition and visual perception, constraints can be employed to identify the spatially proximate and analogous characteristics of housing objects, thereby enhancing the precision of the classification and identification of seismic-resistant structures of houses.

In order to fill the above gaps, this work proposes a method for classifying the structure type of houses based on Gestalt principles for uncrewed aerial vehicle (UAV) remote sensing images. This method classifies the house structures by extracting the features of house objects in UAV remote sensing images and conducting Gestalt clustering analysis. This approach offers a novel technical concept for the study of remote sensing assessment of the seismic capacity of houses. Compared to preceding studies, this study enhances the precision of the classification outcomes by incorporating the spatial distribution characteristics of houses and the attributes of UAV remote sensing images. This approach is designed to address the urban environment’s intricacy and enhance the accuracy of classification outcomes. Consequently, it can be utilized in the seismic capacity evaluation of large-scale building groups.

Despite the advancements made in this study, several limitations still require further investigation. The current study categorizes house structures into three types; however, this classification system is not exhaustive. For instance, Peng Zhou and Yuan Chang employed 29 machine-learning features to classify over 3700 buildings in Beijing into five common structural types [12]. Future research could refine the classification of housing structures by incorporating additional housing attributes. The proposed method was tested on approximately 1600 buildings in a specific area, mainly using images from a UAV remote sensing dataset. This necessitated considerable manual effort to label the experimental data. It is thus recommended that future studies apply this approach to buildings in larger cities, as these areas exhibit diverse functions, forms, and architectural styles.

## 2. Study Site and Study Data

### 2.1. Study Area

**Figure 1:**
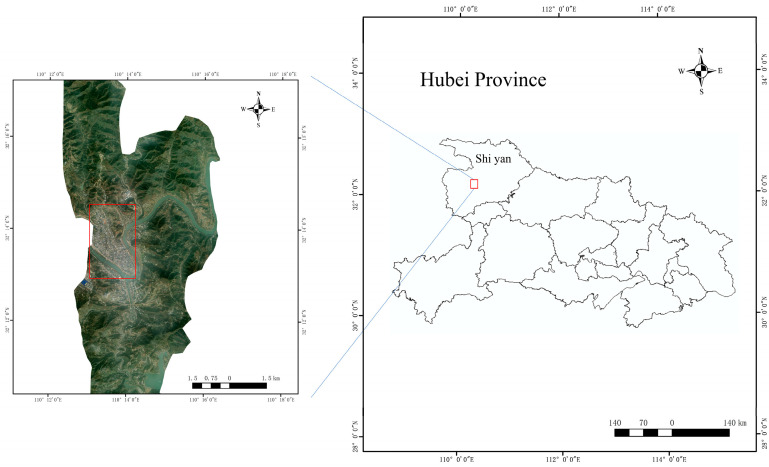
Geographic location map of the study area. The red rectangle is the study area.

This work utilizes the remote sensing classification of building seismic structures in Zhushan County, Shiyan City, Hubei Province, which is the study area. The study area is shown in Figure 1. Zhushan County is situated in the hinterland of the Qinlin-Daba Mountain area in northwestern Hubei Province, within the seismic zone of the middle reaches of the Yangtze River in the seismic zone of South China. The territory encompasses two principal fracture zones, viz., Zhushan and Qingfeng. According to the Fifth Generation of Seismic Ground Motion Parameter Zonation Map of China, the peak acceleration of ground shaking in Zhushan County reaches 0.15 g (g representing the acceleration due to gravity), which is one of the districts and counties with the highest seismic risk in Hubei Province. The residential buildings within the study area can be broadly categorized into the following types: single- or two-story wooden or brick houses, single- or two-story large-span factories, multi-story brick–concrete houses, and multi-story frame houses comprising seven or more stories. The types of building structures in this area are diverse and representative.

### 2.2. UAV Remote Sensing Data

The remote sensing data utilized in this work have been obtained by the DJI M300 UAV, a compact quadcopter equipped with real-time kinematic (RTK) positioning and orientation capabilities. It exhibits a vertical accuracy of ±0.1 m in RTK mode while hovering, and a horizontal accuracy of ±0. The M300 is equipped with a P1 full-frame camera, which is integrated with a full-frame image sensor and a three-axis gimbal. This is paired with the DJI Zhitu for modelling, thus allowing for the operations not subject to image control.

**Figure 2:**
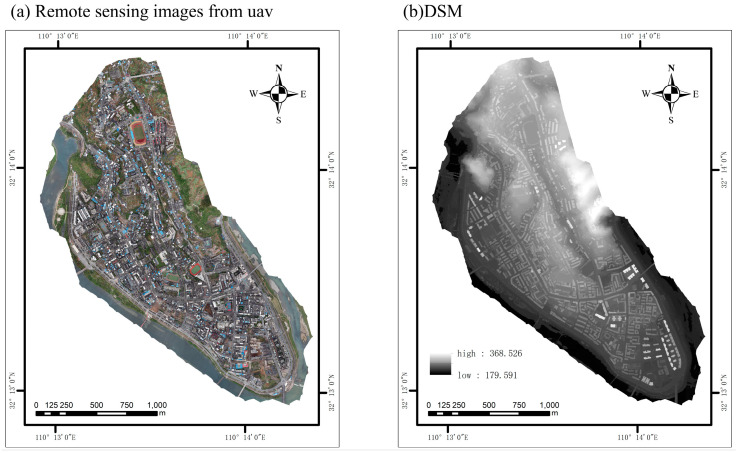
Zhushan County housing profile: (**a**) Zhushan County UAV images, 734 images from DJI M300 RTK; (**b**) DSM images based on UAV remote sensing.

When the UAV collects images of houses in the study area, the aerial photography range is set to be about 3 square kilometers, the shooting altitude to be 400 m, and the overlap rate of the course heading and the side direction to be 70 percent. A total of 734 orthophotos have been collected, and the image reference coordinate system of WGS84. Two-dimensional reconstruction has been carried out on the collected UAV photographs using DJI ZhiTu V4.4.0 software. The spatial resolution of the generated orthophotos and DSMs is about 5 cm. The remote sensing image data set is shown in Figure 2.

### 2.3. Study Area Building Data

**Figure 3:**
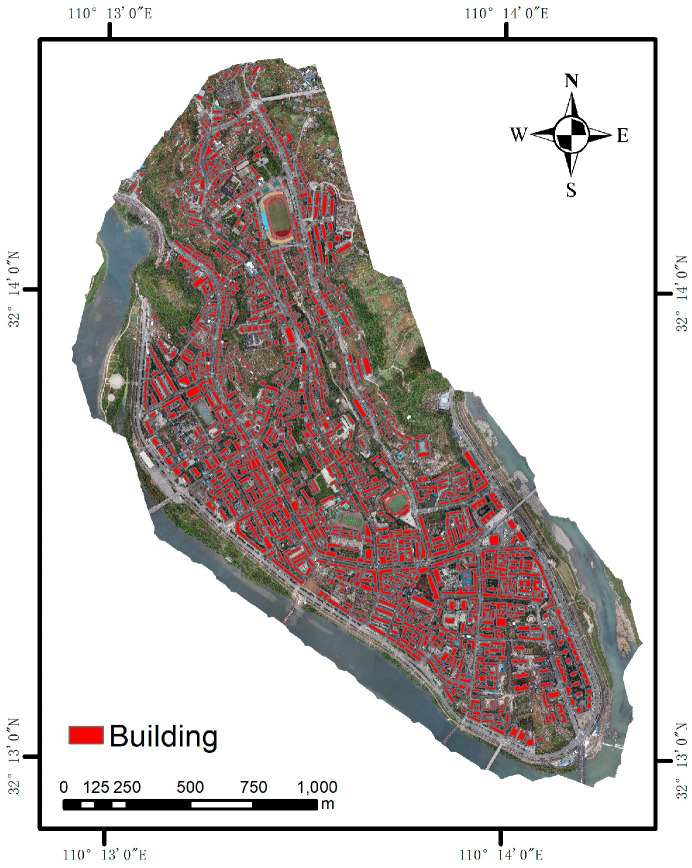
Vector data of houses for classification of house structure types.

To examine the efficacy of a remote sensing classification method for the seismic structures of houses, this work employs a deep learning approach to pre-process the UAV images that are to be collected to extract individual house objects. The house object is extracted using the multi-scale aggregated fully convolutional neural network (MA-FCN) proposed by Zhang et al. [13], with a set number of iterations (60) and a set number of forward/backpropagation samples (8). The resulting extraction is depicted in Figure 3.

## 3. Method

**Figure 4:**
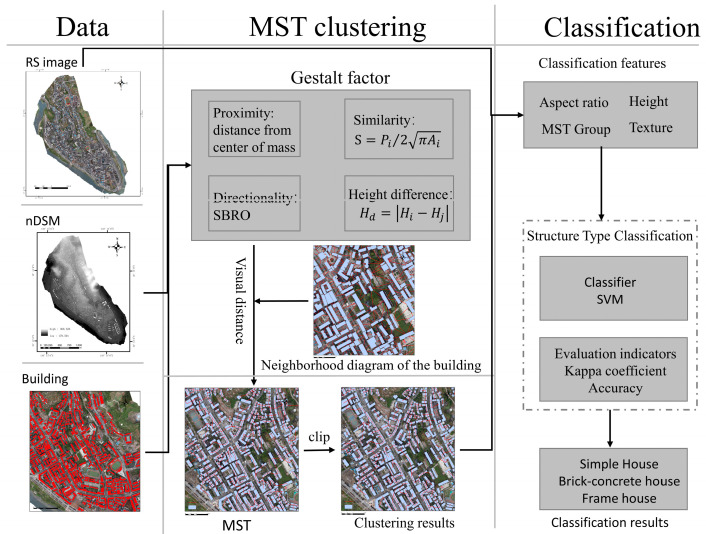
Flowchart of the experiment.

To enhance the precision of house structure type classification, this work proposes a house structure type classification method that is integrated with the minimum spanning tree (MST) clustering, which is based on the tenets of the spatial clustering method. The experimental flow of the method is shown in Figure 4. The method comprises the following two principal stages: MST clustering using UAV remote sensing images and house vector extraction results. (1) The Gestalt factor is calculated through the geometric features of the houses, e.g., for height, area, and shape. Further, the visual distances between the houses are calculated accordingly to quantify the spatial visual similarity, and subsequently, the neighborhood maps between the houses are constructed using the Delaunay triangulation network to reflect the spatial relationships between them. The visual distances and proximity maps are then employed to generate a minimum spanning tree (MST), which is subsequently used to complete the clustering of the houses by cropping the MST. (2) Based on the MST clustering results, the features of the house cluster (e.g., height, texture, aspect ratio, and area) are extracted, and the support vector machine algorithm is applied to classify the houses into three categories of structural types, viz., simple houses, brick–concrete houses, and frame-structured houses.

### 3.1. MST Clustering Based on the Gestalt Principle

The spatial distribution of different structural buildings frequently exhibits spatial autocorrelation, which is influenced by several factors, including natural geography, humanities, economy, and social psychology [14]. This implies that the houses of the same structural type are often distributed in clusters and areas. These houses of a similar structural type are often distributed in clusters and patches, and often reveal similar geometric and textural features upon remote sensing imagery [15,16].

Building clustering is based on the spatial autocorrelation of houses, by employing spatial clustering methods to divide the buildings into separate groups to enhance the similarity of buildings in the same group. The classical spatial clustering methods can be categorized, such as partition-based methods, density-based methods, and graph-based methods [17]. The partition-based method abstracts spatial buildings into point entities and then clusters the point entities, such as through the use of methods based on k-means clustering and predictive modeling to identify clusters of buildings that can be classified by similarity or associated with representative buildings [18]. However, there is a need to identify clusters of buildings that can be classified by similarity or associated with representative buildings; furthermore, partition-based methods are sensitive to the initial parameters, such as the number of clusters, and have a limited performance when dealing with data with complex shapes or significant density changes. Density-based spatial clustering of applications with noise (DBSCAN) is the most representative method, which can automatically identify and eliminate noise points. The performance of DBSCAN depends on the selection of parameters, and its classification accuracy may be affected in the case of high-dimensional data or significant density changes [19,20]. An approach based on graph theory first treats all buildings as a cluster. It is then gradually divided into sub-clusters, such as the minimum spanning tree (MST) pruning method, which uses the minimum distance between buildings to construct an MST, and minimum spanning tree building clusters, by deleting inconsistent edges. This algorithm does not rely on parameter selection, and the internal structure of the data determines the results. It can deal with the distribution of buildings with complex shapes and significant density changes, and is more robust [14]. Therefore, our work employs the minimum spanning tree algorithm for house clustering that groups the dataset by constructing a minimum spanning tree between data points [21]. Employing a minimum spanning tree algorithm for house clustering can eliminate the blindness in clustering and can greatly improve the effect of house clustering.

In the process of housing clustering, peoples’ cognition will be constrained by certain laws and, e.g., the Gestalt visual recognition principle. The Gestalt principle proposes that human beings always observe things with experience, such that there is a certain law that guides the maximization of human perception of the exterior world apart from the whole [22]. Since certain geometric properties of buildings, such as symmetry, closure, and same direction, are exactly in line with certain principles of Gestalt theory, we can utilize three Gestalt factors, viz., proximity, similarity, and directionality among buildings, for the clustering of building groups [23].

#### 3.1.1. Calculation of the Factors of the Gestalt

**Figure 5:**
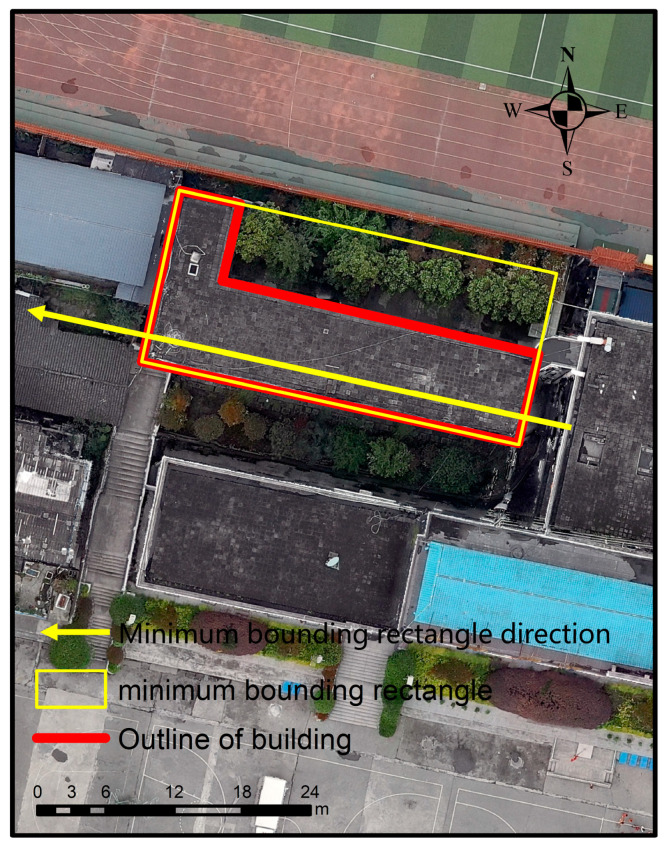
Example of minimum outer rectangle of a building (the bottom image is a remote sensing image of the study area. The red rectangle is the house vector, and the yellow rectangle is the minimum bounding rectangle).

The spatial distance between houses generally represents proximity between houses. Commonly invoked distance metrics include minimum, maximum, and center of mass distance. Since the purpose of this work is to classify the structural types of houses rather than exploring the spatial distribution patterns of houses, the relatively easy to compute center of mass distance has been chosen to represent the proximity between houses. Furthermore, this work employs the closeness S and the minimum area outer rectangle direction D to represent the similarity and directionality between buildings, respectively. The closeness S is defined according to Equation (1), and the minimum area outer rectangle direction S is displayed in Figure 5.

Closeness S

(1) $$S=P_{i}/2\sqrt{\pi A_{i}}$$

where $A_{i}$ and $P_{i}$ represent the area and perimeter of the building, respectively.

The structure type of a house is strongly correlated with the height of the house, and the clustering results obtained by considering the height characteristics of the building are more aligned with the human perception than those obtained by utilizing visual distance solely as a constraint [24]. Our work has loaded the height difference between houses onto the visual distance as a weight to enhance the height difference between two houses, corresponding to the greater visual distance. According to the ‘National Residential Design Code’ (GB50096-2011), the normal residential floor height should be 2.8 m. To avoid the occurrence of two houses having a huge height difference aside from a small visual distance, we take the national housing standard layer three times as $H_{max}$. When the height difference between the houses exceeds thrice the standard value $H_{max}$, the weight is set to a larger value of H. The height difference weights $H_{d}$ are defined as follows:

(2) $$H_{d}=\left|H_{i}-H_{j}\right|$$

(3) $$\left\{\begin{array}{c} H_{d}=H_{d}\;\;\;\;\;\;H_{d}<H_{max}\\ \;\;H_{d}=H\;\;\;\;H_{d}>H_{\max} \end{array}\right.$$

#### 3.1.2. Building Neighborhood Diagram Generation

**Figure 6:**
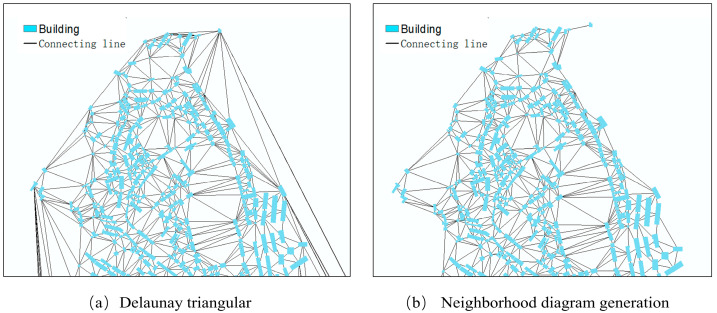
Example of house neighborhood diagram generation: (**a**) Delaunay triangular grid generated based on house point data; (**b**) Delaunay triangular mesh for neighborhood diagram generation of houses.

The nodes and edges of the house neighborhood graph represent the buildings and the neighborhood-related undirected graph between buildings, respectively. In this study, Delaunay triangular meshes have been constructed through the centroids of the house polygons to connect the houses into a triangular mesh without overlapping, and Delaunay triangular meshes have the nearest neighbor connectivity property, which can accurately analyze the proximity relationships between spatial objects [25]. However, the constructed triangular mesh may contain edges with excessive edge lengths induced by the data noise or uneven distribution of buildings. To obtain an accurate proximity map, it is necessary to exclude the triangles with abnormal side lengths at the periphery by setting a threshold. The neighborhood graph creation process is shown in Figure 6.

#### 3.1.3. Constructing the MST

The main algorithms for generating MST are the methods of circle avoidance [26] and edge cut [27], among which the Prim algorithm is suitable for computing minimum spanning trees for dense points. It can construct the minimum spanning tree by vertex-by-vertex connectivity, which is more suitable for house clustering. In a given neighborhood diagram $G=\left(V,E\right)$, $\left(u,v\right)$ represents the edge connecting vertex u to vertex v. $W\left(u,v\right)$ represents the weight of this edge, and if *T* exists, which is a subset of E and is an acyclic graph, then $W\left(T\right)$ such that *G* has the smallest $W\left(T\right)$. Then, T is the minimum spanning tree of G. The visual distance-based MST is obtained by assigning visual distances to $W\left(u,v\right)$ as weights for MST generation.

(4) $$W\left(T\right)=\sum_{\left(u,v\right)\in T}W\left(u,v\right)$$

The visual distance is defined by the composite of the center of mass distance, direction difference, size difference, and height difference, where $C_{s}$, $C_{d}$ represent the weights of the direction difference and size difference, respectively [28]. The range of variation of the set weights is [1, 2], $C_{d}$ corresponds to the direction angle of [0, 90°], and the size difference is expressed by the area ratio (by the small area over the large one in it). The range of variation $C_{s}$ is [1, 2], corresponding to an area ratio of [1, 0.25], and if the area ratio is below 0.25, the weight of the size difference is fixed as 2. The specific formula is defined as follows:

(5) $$V_{d}=f\left(cen_{dis},C_{d},C_{s},H_{d}\right)$$

(6) $$C_{d}=\left|D_{i}-D_{j}\right|+1$$

(7) $$C_{s}=2-\frac{\min\left(S_{i}\;,S_{j}\right)}{\max\left(S_{i}\;,S_{j}\right)}$$

(8) $$\left\{\begin{array}{c} C_{s}=C_{s}\;\;\;\;\;C_{s}<1.75\\ {\;\;C}_{s}=2\;\;\;\;\;\;C_{s}>1.75\;\; \end{array}\right.$$

(9) $$C_{d}=\left|D_{i}-D_{j}\right|+1$$

#### 3.1.4. MST Cutting

Pruning any edge of MST will partition the target group into two, and different levels of clustering results can be obtained by setting a threshold for pruning MST. Zahn [29] has identified the problem of ‘inconsistent edges’ in MSTs whose weights are much larger than the average weights of the edges of MST. Further, a threshold can be set to prune these ‘inconsistent edges’ to obtain reasonable clustering results. The appropriate threshold setting, also known as the optimal number of clusters, is one of the crucial factors in determining the classification accuracy of building structure types. When the number of clusters formed by the building clustering is around 20% of the number of buildings, the clustering results are in accordance with human visual cognition. However, the main research of this work is on the classification of building structure types, rather than the spatial distribution patterns of buildings. Therefore, the number of clusters formed by the building clustering has been increased to reduce the number of clusters with structure type clustering errors. Taking the number of clusters of the wrong building structure clustering as the evaluation index, the number of clusters formed by building clustering is 10%, 15%, 20%, 25%, 30%, and 35% of the total number of buildings by setting the threshold; comparative analysis determined that the optimal threshold cluster number was 25% of the total number of buildings. Section 3.2 gives the specific analysis of the number of clusters with structural-type clustering errors.

### 3.2. Texture Feature Extraction

**Figure 7:**
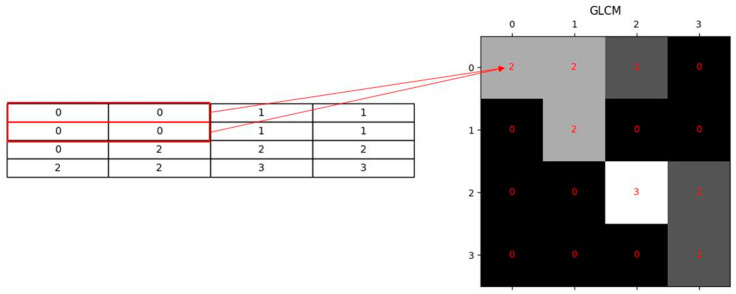
Conversion of grayscale map to GLCM (direction 0°, distance 1).

Our work has texture features extracted from UAV remote sensing images using the Gray Level Covariance Matrix (GLCM). Further, the work has first proposed to extract texture information from images by calculating the spatial relationship between pixels [30]. The GLCM is defined in terms of the joint probability density of pixels at two positions $P\left(\left(i,j\right)/\left(d,\theta \right)\right)$, where $P\left(\left(i,j\right)/\left(d,\theta \right)\right)$ denotes the probability of occurrence of a pair of image elements with gray values i and j, respectively. They are separated by a distance of d image elements in the θ direction, whose order is determined by the gray level $N_{g}$ in the image. Figure 7 depicts the computational principle of the GLCM with the image element distance d as 1 and the direction θ = 0. The gray level of the image is denoted on the left side. The smallest, largest, and the $N_{g}$ of the gray levels are 0, 3, and 4, respectively, and the corresponding gray covariance matrix is shown on the right. The GLCM reflects the distribution characteristics of luminance in addition to the position distribution characteristics between pixels with the same or similar luminance, which is a second-order statistical feature regarding the luminance change of the image.

After calculating the GLCM, obtaining the texture features used for image classification is not intuitive. Calculating the texture feature statistics is necessarily based on the gray level covariance matrix. Haralick has proposed 14 kinds of feature statistics calculated by the GLCM. Ulaby, et al. [31] have found that among the 14 texture features proposed by Haralick, only contrast, entropy, correlation, and energy four feature statistics are irrelevant, owing to which the computational efficiency is reduced and the classification accuracy is also improved. Assuming that the gray level of the remote sensing image is N, $P\left(i,j\right)$ denotes the value at the position $\left(i,j\right)$ in the GLCM, μ at the mean of the gray covariance matrix, σ at the variance of the gray covariance matrix, and the definitions of the four feature statistics values are shown below.

1. Contrast

(10) $$con=\sum_{i=0}^{N}\sum_{j=0}^{N}{\left(i-j\right)}^{2}P\left(i,j\right)$$

2. Correlation

(11) $$cor=\sum_{i=0}^{N}\sum_{j=0}^{N}\frac{\left(i-\mu \right)\left(j-\mu \right)P\left(i,j\right)}{\sigma^{2}}$$

3. Angular second moment

(12) $$ene=\sum_{i=0}^{N}\sum_{j=0}^{N}{P\left(i,j\right)}^{2}$$

4. Energy

(13) $$ent=\sum_{i=0}^{N}\sum_{j=0}^{N}{\left(i-j\right)}^{2}logP\left(i,j\right)$$

### 3.3. Classification of Housing Structure Types

#### 3.3.1. Standards for the Classification of Structure Types

**Figure 8:**
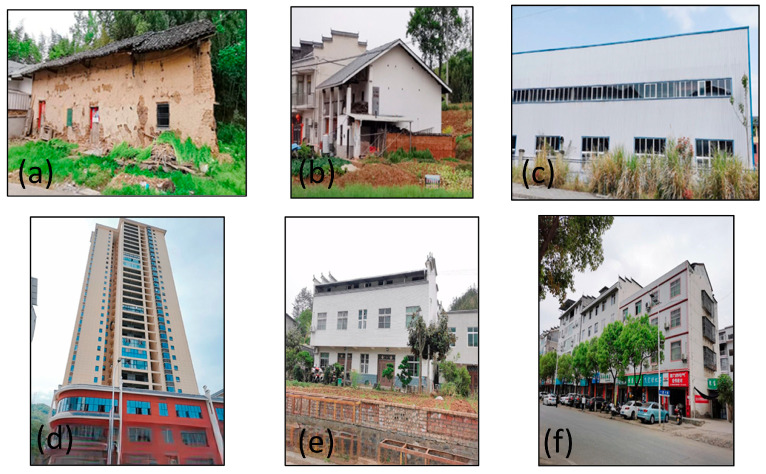
Schematic diagram of typical house structure types in Zhushan County. (**a**) wooden or brick houses; (**b**) multi-story brick–concrete houses; (**c**) single- or two-story large-span factories; (**d**) multi-story frame houses comprising seven or more stories; (**e**,**f**) multi-story brick–concrete houses.

**Figure 9:**
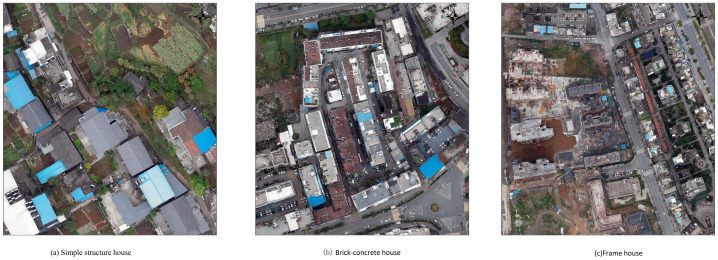
Example diagram of classification structure.

The seismic performance of houses strongly correlates with their structural types. By summarizing the correspondence between the image features and the actual features, and combining them with the standards for assessing the seismic capacity of houses, this study simplifies the structural type of houses into the following three categories: simple, brick–concrete, and frame houses. The remote sensing characteristics of each type of houses are as follows: simple houses are smaller in area, darker in roof color, with one to three floors, and usually of civil engineering, brick, and steel structure. Their building structure type characteristics are shown in Figure 8a–c, and the remote sensing image characteristics are shown in Figure 9. Brick and concrete houses have a large single area, different roof textures, and 3–7 stories, thus forming a regular building complex. Their building structure type features and remote sensing image features are illustrated in Figure 8d,e and Figure 9b, respectively. Brick and concrete houses are more diverse and can easily be misclassified with other house types in the classification process. Frame houses are large in area, symmetrical in shape, with light-colored roofs and a high number of stories, and are usually distributed in clusters. Their building structure type characteristics are shown in Figure 8f and the remote sensing image characteristics are shown in Figure 9c.

#### 3.3.2. Classification of Structure Types

This work employs a support vector machine (SVM) to classify building clusters generated by clustering houses by using the mean of features such as texture, height, and house area of the building clusters. SVM is a machine-learning method based on statistical learning theory and the principle of structural risk minimization proposed by Cortes and Vladimir [32]. It is suitable for small sample problems. The basic principle of SVM is to identify a hyperplane in the high-dimensional space that maximizes the distance between the data points of different categories to achieve effective classification [33]. There are two main points in the basic idea of SVM, as follows: (1) Based on the theory of structural risk minimization, we find the optimal hyperplane in the feature space of samples to maximize the interval between positive and negative samples. (2) For the linearly indivisible problem, a nonlinear mapping algorithm has been generally employed to transform the samples that are linearly indivisible in the low-dimensional space into the high-dimensional feature space to make them linearly divisible.

This paper selects the RBF kernel as the inner product kernel function. The RBF kernel has a strong nonlinear mapping ability, can effectively deal with complex classification problems, and is adaptable to high-dimensional data. The advantages of RBF kernels include strong sensitivity to parameter adjustment and good robustness, especially in the case of small datasets or considerable noise, which make RBF kernels generally more stable. Compared to other commonly used kernel functions, such as linear kernel, sigmoid kernel, and polynomial kernel, the RBF kernel usually performs better when dealing with nonlinear data [34].

The SVM is a typical two-class classifier, which only determines whether a sample belongs to a positive or negative sample. However, the problems to be solved in practical applications are often multi-classification problems. The employment of SVM for multi-classification must be divided into several binary classification problems. Each binary classification problem is being utilized to build an SVM classifier, and the combination of these SVM classifiers are used to solve the multi-classification problem. There are two main ideas, which are as follows: one-versus-many and one-versus-one [35]. In the one-versus-many method, each category features as a positive set by extracting the training set, and the rest of the categories are a negative set, which will be used as the sample. The number of categories of the same classifier to classify the prediction do not apply to the data imbalance situation. The one-to-one method is implemented by designing a classifier between any two categories of samples. The first SVM classifier is constructed by taking the first category samples as positive samples and the second category samples as negative samples. Then, the second SVM classifier is constructed by taking the first category of samples as positive samples and the third category of samples as negative samples, until an SVM classifier is constructed between every two samples.

## 4. Experimental Results

### 4.1. MST Clustering

**Figure 10:**
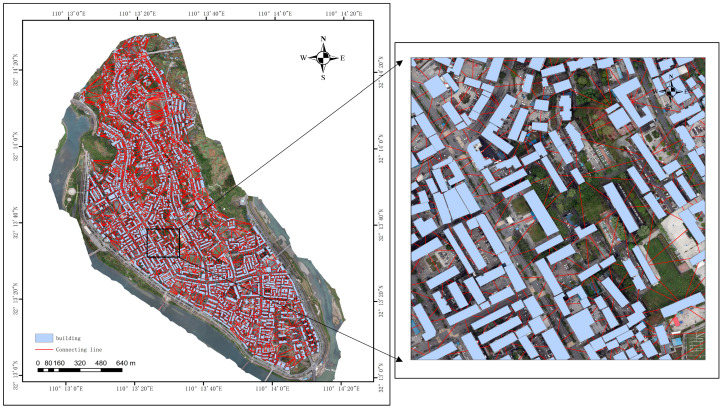
Neighborhood diagram of houses in the study area (the picture on the right shows the details).

**Figure 11:**
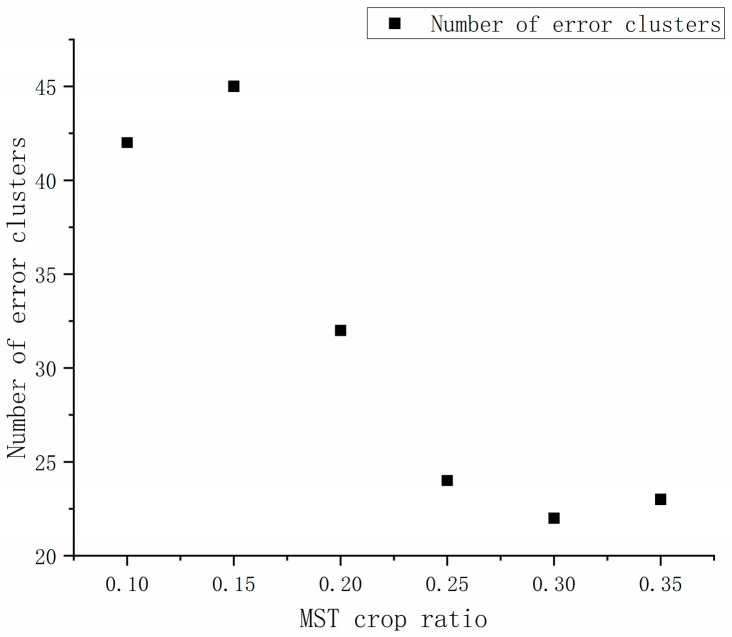
Analysis of clustering results for a different number of clusters (the abscissa is the ratio of the number of clusters to the total number of buildings, and the ordinate is the number of wrong clusters).

**Figure 12:**
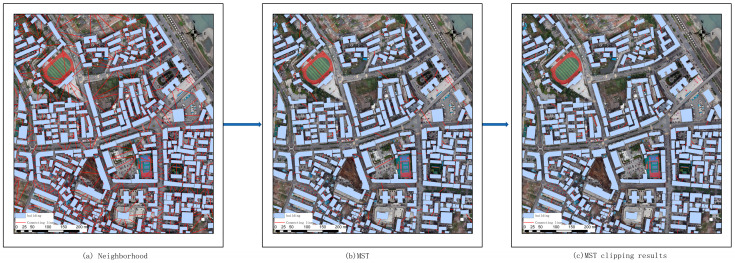
MST clustering and cropping results.

(1) We employ ArcGIS 10.3 to generate a Delaunay triangle mesh based on the center of mass of the house and then delete the triangles in the periphery whose side lengths exceed a certain distance to derive the neighborhood diagram generation of the house, and the results are shown in Figure 10.

(2) Calculate the tightness S and the minimum area outside the rectangle direction D. The results of these calculations are listed in Table 1. The direction, size, and height differences are to be calculated by Equations (6)–(9). The resulting values are listed in Table 2, where Building_1 and Building_2 represent the house numbers connected by the nearest connecting lines in the diagram.

(3) To identify an appropriate threshold for determining the optimal number of clusters, this work employs the number of clusters erroneously assigned to the incorrect house structure as an evaluation metric. The objective is to set the threshold at a level which results in forming a single cluster comprising the clustered buildings. The optimal threshold for clustering is determined by comparing and analyzing the number of houses in each category, which includes 0%, 15%, 20%, 25%, 30%, and 35% of the total number of houses. The specific results are depicted in Figure 11. As illustrated in the clustering results graph, an increase in the number of clusters formed in the total number of houses before 25% can effectively reduce the number of error clusters. However, beyond this threshold, the number of error clusters remains relatively constant. Further, the formation of additional clusters leads to an increase in the amount of computation and the number of detached buildings. Accordingly, we employ a threshold of 25% of the total number of houses in the clusters formed by the house clustering, in conjunction with a visual distance threshold of 40, for the purpose of MST cropping. The Prim algorithm has been employed to crop the MST, resulting in the generation of 430 groups of house clusters. The clustering process and results are illustrated in Figure 12.

### 4.2. Texture Feature Extraction Result

Several commercial or open-source pieces of software can extract image texture features, and we employ eCognition for texture feature extraction. The UAV remote sensing image and the house vector shape file are imported into the system, and the UAV image is preliminarily segmented according to the house shape file to extract the house part. Owing to the considerable size of the remaining non-house part, it is challenging to compute, thus necessitating a second segmentation to enhance computation speed. Ultimately, the texture features are calculated and exported, and the results of texture feature extraction are listed in Table 3.

### 4.3. Classification of Structural Types

**Figure 13:**
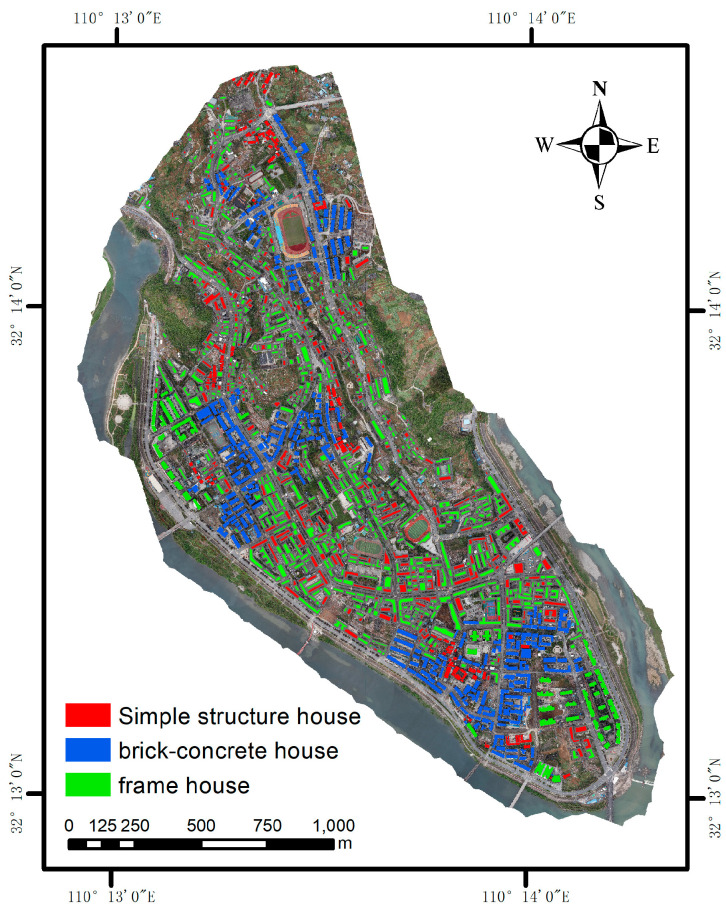
Results of classification of building structure types (the red area is simple houses, the blue area is brick house, and the green area is frame house).

This work annotates the houses from the classification of house structure types presented in Section 3.3.1, with the additional input of the field survey and visual interpretation. Subsequently, a support vector machine (SVM) has been employed to classify house structure types, with the penalty parameter C of the SVM set to three. When the penalty parameter C of the SVM increases, the penalty for misclassification also rises. The degree of fitting to the training set can be interpreted as the value of C. A smaller value of C will result in more smooth decision boundaries. In contrast, a larger value of C will result in the decision boundaries being more focused on a small number of sample points. The kernel function employs a Gaussian kernel, with housing structures classified as simple, brick, or frame houses. The sample category is relatively limited, and the distribution of each structure type of urban housing is not uniform. Furthermore, the sample data are not balanced. A one-to-one classification method has been employed for SVM classification to address these issues. This method can circumvent the indivisibility caused by the limited sample category. The initial 100 groups of house clusters, comprising 584 buildings, have been selected as the training set. The final classification results are presented in Figure 13, with the red, blue, and green houses representing simple, brick, and frame houses, respectively. 

### 4.4. Validation of the Results

**Figure 14:**
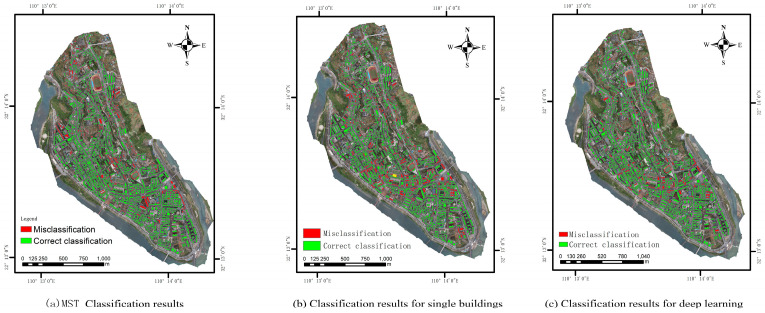
Comparison of results of different classification methods (overlay comparison with field survey data).

In order to make a comparative analysis of the methods used in this paper, this study uses the remote sensing preliminary judgment method of building seismic capacity based on a deep learning remote sensing target recognition algorithm and multi-scale aggregation proposed by [13], and the classification of building structure types based on a single building are used to classify the research image. The overall accuracy of the remote sensing initial judgment of brick–concrete houses by deep learning is about 90.9%, about 72.3% for simple houses, and about 85.4% for frame-structured houses. The overall accuracy of the remote sensing initial judgment of brick–concrete houses based on single-family building structure types is about 96%, about 35% for simple houses, and about 84% for frame-structured houses. The comparison of classification results is shown in Figure 14.

The classification results of building structure types based on MST clustering are shown in Table 4. The method used in this paper considers the spatial similarity between buildings, and the classification accuracy of brick–concrete buildings is improved to 95.4%, that of simple houses is improved to 93.4%, and that of frame-structure buildings is improved to 87%. The Kappa coefficient is increased to 0.89, and the consistency between predicted and actual results is significantly improved. By using MST clustering to classify buildings with the same structure into buildings and using the average value of the characteristics of the buildings to classify the structure types, the influence of similar characteristics between buildings with different structures on the classification results can be effectively reduced. According to the classification results of building structures in Table 4, 499 buildings are simple houses, of which 40 buildings are wrongly classified as brick–concrete buildings; 644 are brick–concrete buildings, of which 22 buildings are wrongly classified as simple houses and 5 buildings are wrongly classified as frame structures; 33 houses are frame structures, and no other houses with a frame structure have been wrongly classified. The results of different classification methods are shown in Table 5.

## 5. Analysis and Discussion

### 5.1. Effectiveness of MST Clustering-Based Classification of Housing Structure Types

To conduct a qualitative analysis of the classification results, we employ a kernel density estimation to examine the distributional characteristics of houses in Zhushan County. Utilizing a dynamic cell, kernel density analysis enables the description of point density around each image element, thereby generating a density heat map [36]. This methodology facilitates the analysis of the distribution of research elements within the study area, thus quantifying the degree of density. Furthermore, it enables a visual analysis of the geographical distribution of elements of the law. The nuclear density analysis has two primary functions, which are as follows: firstly, it facilitates comprehension of the spatial distribution of houses, and secondly, it provides a valuable reference point for urban planning and disaster prevention. In urban planning, kernel density analysis can reveal the thermal areas in which houses are distributed, thereby assisting planners in identifying too-dense areas. Concerning disaster prevention, the seismic capacity of buildings is related to the density of their spatial distribution. For instance, Qin et al. [37] proposed a refined assessment framework of urban disaster vulnerability based on risk factors and calculated the spatial distribution of the exposure index by using kernel density analysis combined with the spatial point data of risk urban factors.

**Figure 15:**
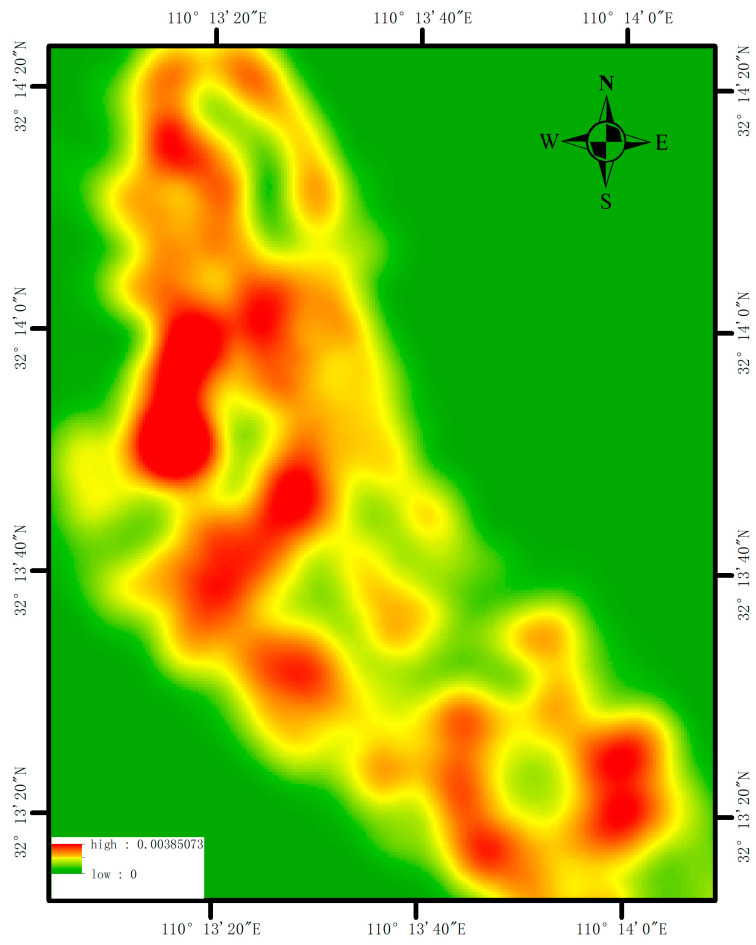
Thermogram of spatial distribution density of houses.

The resulting specifics of the kernel density map of the spatial distribution of houses in Zhushan County are listed in Figure 15. The density thermogram of houses in Zhushan County reveals a relatively uniform overall distribution, with the presence of multiple high-density clusters of buildings. Owing to the time progress, the distribution of houses has evolved from a dense configuration to a more rational layout, thus accompanied by a notable reduction in density. As a general rule, older houses are distributed more densely, and most houses in the area are simple or brick–concrete structures. The red color on the heat map indicates these features, whereas frame structures are distributed more sparsely. For the area of the single unit, the houses have larger spacing, and the spatial distribution is sparse, which corresponds to the newly-built houses. These are distributed in the city’s peripheral areas, i.e., the green area of the heat map. The spatial distribution characteristics of housing structure types are consistent with the classification basis of seismic capacity and the remote sensing characteristics of houses in the classification basis of structure types. Overall, the frame houses are distributed sparsely, whereas the brick–concrete and simple houses are widely distributed with a high density.

### 5.2. The Clusterability of MST Clustering

The results from the house clustering demonstrate that the target sets with discernible group structures can be clustered together. This is largely consistent with the visual recognition that an uninformed observer would make. The simple houses and the surrounding brick–concrete houses are effectively clustered in the corresponding clusters, which demonstrates that the MST clustering is capable of accurately identifying and categorizing houses with similar characteristics. Further, it provides clustering results that are consistent with the actual structure of the building clusters. However, the accuracy of the clustering results may be compromised in instances where the dimensions of the edifices exhibit considerable disparity and their disposition is irregular. Further, the distinction between structures with multiple interconnected edges and their neighboring edifices within the building clusters is pronounced. This indicates that the efficacy of the MST clustering method may be constrained in this intricate context. The question of defining the priority of each Gestalt factor and judging the degree of influence of each factor on the clustering results, in addition to giving different weights to different Gestalt factor to obtain more accurate clustering results, remains to be addressed in greater depth. Furthermore, only one factor, viz., building height, has been employed in this experiment, thus incorporating additional semantic features of buildings that could enhance the efficacy of MST clustering, whereas there is scope for enhancement in the accuracy of MST clustering in certain complex scenarios; thus, the method is generally effective in accurately clustering houses with the same structure type.

### 5.3. Classification Results of Application Meaning

The results of the classification of house structure types in this study are of tremendous significance in assessing the seismic capacity of houses. The seismic capacity of a building is intimately related to the type of structure it is constructed from, according to Sun and Yan [38]. Furthermore, it is possible to make an informed estimation of its seismic performance by classifying the structure of a building. For instance, the masonry walls and columns of simple structure houses lack sufficient strength, and most of these structures are advanced in age. Consequently, they are susceptible to damage on the roof and partial collapse in earthquakes. The classification of house structures enables the formulation of reinforcement and retrofitting programs for old buildings or houses in seismically susceptible areas. This is to enhance their seismic capacity and reduce the losses caused by seismic hazards.

The Expert Group on Civil Engineering Structures of Tsinghua University, Southwest Jiaotong University, and Beijing Jiaotong University have conducted statistical analyses of earthquake damage conditions on the building damage data investigated in the main disaster areas of the Wenchuan Earthquake in Sichuan Province in 2008. The results of this analysis have indicated that the construction age of a building also has a certain influence on its seismic capacity [39]. Zhu et al. [40] have proposed a method for inferring the age of houses through the point density index of remote sensing image interpretation units; however, the method manually divides the building groups for point density calculation. We have classified the structure type of houses using MST clustering, which can automatically calculate the point density of house clusters. This allows the age of houses to be ascertained.

## 6. Conclusions

The objective of this work is to conduct a comprehensive evaluation of the seismic resilience of building structures. It introduces a novel approach to spatial clustering based on the original method to enhance the accuracy of the classification of building structures. This work studied the classification of house building structure types from UAV remote sensing images, with the houses in Zhushan County, Hubei Province, serving as the case study. Based on a synthesis of the findings from previous works, we propose a methodology for classifying the house structure types based on UAV remote sensing images and MST clustering. We employ a support vector machine to classify the house structure types by using the geometrical, textural, height, and spatial distribution of UAV remote sensing images of the house clusters with multiple features. Attributes have been used to classify house types, including simple, brick, and frame structures. The accuracy of the classification results has been evaluated by combining the field survey and visual interpretation results, thus resulting in an overall accuracy of 94.3%, with a Kappa value of 0.89. These findings support the feasibility of using MST clustering for classifying the house structure types. In addition to improving the classification accuracy, the method proposed in this paper has a far-reaching impact on urban planning and seismic capacity. In urban planning, accurately classifying the spatial distribution of houses with different structural types can provide data support for urban earthquake-resistant planning and optimal allocation of resources. In the aspect of seismic capacity, this method can help evaluate the seismic capacity of different building structures and provide a scientific basis for the seismic capacity of buildings.

Three avenues of future research may be pursued to enhance the methodology as proposed in this paper. (1) To describe the geometric features of the buildings, it is necessary to define the priority of each Gestalt factor and judge the degree of influence of each factor on the clustering results. (2) The classification accuracy of SVM is critically influenced by its parameter selection, particularly the type of kernel function. Since SVM has evolved, a greater range of kernel functions and parameters has become available, thus increasing the complexity of the model selection process and the computational burden. Future research can further optimize the parameter selection process of SVM. (3) The disparate spatial distribution of residential properties across various geographical regions, particularly in historic urban areas or cities exhibiting pronounced variations in distribution, can potentially compromise the efficacy of classification methodologies. The configuration and structural attributes of edifices within these regions can vary considerably, thereby precipitating inconsistencies in the outcomes of classification processes. Consequently, optimizing these discrepancies assumes considerable significance as a research agenda for future studies.

## Figures and Tables

**Table 1:** Results of house Gestalt factors (some examples).

	Tightness	SBRO
1	0.004151	28.61042
2	0.126313	57.65
3	0.019862	33.85711
4	0.453388	63.43
5	0.024835	28.44293
6	0.100902	86.14936
7	0.041651	3.29
8	0.033608	36.25384
9	0.034374	24.17911
10	0.017542	33.29942

**Table 2:** Relevant parameters for visual distance calculation (some examples).

	Building_1	Building_2	Size Differences	Direction Differences	Height Difference	Centroid Distance
1	1224	1669	1.42	1.87	0.6	190.22
2	1227	1224	2	1.24	6.86	54.13
3	1669	1227	1.72	1.43	6.26	165.85
4	1224	1225	1.52	1.22	2.09	2.72
5	1225	1669	1.2	1.41	1.49	166.54
6	1216	1225	1.05	1.01	0.82	97.1
7	1224	1216	1.55	1.21	2.92	122.43
8	1216	1217	1.5	1.01	15.01	44.24
9	1217	1225	1.51	1.03	14.23	56.38
10	1660	1217	2	1.46	18.37	221.37

**Table 3:** Texture feature extraction results (some examples).

	GLCM_Cor	GLCM_Con	GLCM_Ene	GLCM_Ent
1	0.992759	30.20735	0.003079	6.507599
2	0.979836	69.11691	0.001539	7.329166
3	0.936264	135.5542	0.005983	5.98794
4	0.957779	127.4003	0.00065	7.992059
5	0.934181	132.1597	0.003251	6.386413
6	0.975331	102.5023	0.003143	7.148606
7	0.855336	424.5163	0.000643	8.064305
8	0.85708	394.04	0.00047	8.295183
9	0.919876	171.1447	0.001127	7.360811
10	0.922893	245.4016	0.000477	8.155952

**Table 4:** Confusion matrix and kappa coefficient calculation for housing structure types based on MST clustering.

	Simple Structure	Brick–Concrete House	Frame House	Total
Simple structure	459	40	0	499
Brick–concrete house	22	617	5	644
Frame house	0	0	33	33
Total	481	657	38	1176
kappa coefficient	0.89	Total accuracy	94.3%	

**Table 5:** Comparison of results of different classification methods.

	Simple Structure	Brick–Concrete House	Frame House
Deep learning for classification of building structure types	90.9%	72.3%	85.4%
Classification of structure types based on single building	35%	96%	84%
Classification of building structure types based on MST clustering	95.4%	93.4%	87%

## Data Availability

Dataset available upon request from the authors.
